# AI research trends in liver cancer bibliometric analysis

**DOI:** 10.1097/MD.0000000000046891

**Published:** 2026-01-02

**Authors:** Tingxuan Zhang, Youhui Zheng, Chi Zhang, Weidong Li, Zhanjin Wang, Kaihao Du, Zhan Wang

**Affiliations:** a Affiliated Hospital of Qinghai University, Xining, China.

**Keywords:** AI, artificial intelligence, bibliometric analysis, liver cancer, research trends

## Abstract

**Background:**

The pathogenesis of liver cancer is complex, leading to poor prognosis. Early diagnosis and metastasis monitoring are crucial. With advancements in medical concepts and technologies, this field faces challenges and opportunities. This study explores the progress, hot topics, and future trends of artificial intelligence (AI) in liver cancer diagnosis and treatment. Using bibliometric methods, a comprehensive report on AI in liver diseases is compiled for researchers and practitioners.

**Methods:**

The Web of Science Core Collection database was used to retrieve AI-related liver cancer research literature from 2005 to 2024. Analytical tools like VOSviewer, Citespace, and RStudio were used for bibliometric analysis and knowledge map construction.

**Results:**

A total of 2922 papers were collected in this study, including 2607 original research articles and 315 review documents. In terms of publication volume, China leads the way, while the United States exhibits significant influence in this field with the highest h-index and total citation count. At the institutional level, the top 3 most productive institutions are the University of California System, Harvard University, and the University of London. Regarding author contributions, Loomba, Rohit is the author with the highest number of published papers, while Younossi, Z.M. has the highest number of co-citations. At the journal level, Scientific Reports and Hepatology rank first in terms of the number of published papers and co-citations, respectively, reflecting their importance and influence in this field. From the collected literature, 4419 keywords were extracted, with 119 appearing >20 times. Clustering analysis revealed 3 major clusters. Frequent keywords included “classification,” “diagnosis,” “survival,” and “prediction,” highlighting current research hotspots. Computed tomography is the most common data type used in AI liver cancer research, followed by magnetic resonance imaging and ultrasonography.

**Conclusion:**

Research on AI in liver cancer is still exploratory. With rapid AI advancements, its applications in diagnosis, treatment, and prevention are growing. This bibliometric study aims to analyze the current status of AI in liver cancer research, revealing potential directions and hotspots for further exploration and development.

## 1. Introduction

Liver cancer ranks as the 5th most common cancer among males and the 6th among females globally, and it is also the 3rd leading cause of cancer-related deaths worldwide. Among liver cancers, hepatocellular carcinoma (HCC) accounts for approximately 90% of all cases, with over 500,000 new cases reported annually.^[1]^ Despite some progress in the diagnosis and treatment of liver cancer, such as improved accuracy in diagnostic imaging techniques and enhanced survival rates following neoadjuvant or conversion therapies,^[2,3]^ these advancements remain limited and do not fully meet clinical needs. Therefore, accurately screening early-stage liver cancer patients and high-risk populations, as well as formulating reasonable treatment strategies for advanced liver cancer patients, have become urgent issues to be addressed. The introduction of artificial intelligence (AI) technology provides new insights into solving these problems.

As an important branch of computer science, AI strives to simulate human brain functions and intelligent behaviors, encompassing a series of complex processes such as learning, reasoning, thinking, and planning.^[4]^ With the continuous accumulation of medical big data and the rapid development of computer technology, AI technologies based on machine learning and deep learning have been widely applied in medical research.^[4–8]^ These technologies, leveraging their self-learning, data summarization, and induction capabilities, can construct intelligent reasoning systems to optimize clinical decision-making. Especially in the diagnosis of liver cancer, given the pivotal role and increasing standardization of imaging diagnosis, AI research based on imaging has emerged. These studies can extract high-throughput features from massive image data that are difficult to capture by human vision, thereby constructing intelligent decision-making models to assist in clinical decision-making.^[9–11]^ Specifically, using advanced AI technologies such as artificial neural networks and convolutional neural networks (CNN) to conduct in-depth analysis of medical images such as endoscopic ultrasound and computed tomography (CT) can significantly improve the accuracy of cancer diagnosis and effectively reduce the interference of human experience and subjective factors on diagnostic outcomes.^[12–14]^

To gain a deeper understanding of the application and development trends of AI technologies in the field of liver cancer, we employed bibliometrics, a scientific method for analysis. Bibliometrics is a scientific approach to assessing and monitoring the development trends of specific disciplines through statistical analysis of published literature.^[15]^ It quantifies the research output and citation frequencies of countries, institutions, and authors, and reveals hot topics and frontier trends within specific research areas through keyword frequency analysis. This study aims to provide a comprehensive overview of the evolution of scientific literature in the application of AI technologies in liver disease research from 2005 to 2024 using bibliometric techniques. During the literature analysis process, we conducted a systematic evaluation based on the following criteria: publication year, country distribution, affiliated institutions, journal sources, author contributions, keyword usage, citation networks, and H-index and other academic impact indicators.^[16]^ These criteria collectively constitute an analytical framework, enabling us to comprehensively and deeply understand the development trajectory of this field. This comprehensive analysis method not only provides researchers with rich data support and powerful tool references but also offers useful guidance for them to formulate research plans and decision-making processes. By combining bibliometric analysis results with insights based on traditional review methods, this study not only demonstrates the historical evolution and current state of AI technology research in the field of liver cancer but also further explores future research directions and potential trends.

## 2. Methods

The data utilized in this study were sourced from the Web of Science Core Collection (WoSCC) database of the Science Citation Index Expanded and collected on September 1, 2024. The specific search criteria were set as follows: TS = (artificial intelligence OR “computational intelligence” OR “deep learning” OR “computer aided” OR “machine learning” OR “support vector machine” OR “data learning” OR “artificial neural network” OR “digital image” OR “convolutional neural network” OR “evolutionary algorithms” OR “feature learning” OR “reinforcement learning” OR “big data” OR “image segmentation” OR “hybrid intelligent system” OR “recurrent neural network” OR “natural language processing” OR “bayesian network” OR “bayesian learning” OR “random forest” OR “multiagent system”) AND TS = (liver OR hepatic) AND TS = (neoplasm OR cancer OR tumor OR oncology OR carcinoma OR adenocarcinoma). In this study, we focused on the 2 document types of “articles” and “reviews,” with a time range set from “2005 to 2024,” and only literature published in the “English” language was selected. The search results were saved in both “Plain Text File” and “Full Record and Cited References” formats to ensure the comprehensiveness and accessibility of the data.

Subsequently, we systematically extracted the following basic information from each selected literature piece as the foundation for subsequent data analysis: Country, Author/s, Institution/s, Journal Title, Reference List, and Keywords. This information collectively constitutes the core component of the dataset for this study, providing a solid basis for in-depth exploration and analysis of the current application status, development trends, and collaboration networks of AI technologies in liver diseases.

For the visual analysis of bibliometrics, we employed tools such as VOSviewer (version 1.6.20; Centre for Science and Technology Studies [CWTS], Leiden University, Leiden, Netherlands), Citespace (version 6.3.R1; Drexel University, Philadelphia), and RStudio (version 4.3.3; RStudio, Inc, Boston).^[17–19]^ Specifically, we utilized VOSviewer and Citespace software to conduct visual analysis of the collaboration networks among countries/regions, institutions, journals, authors, and cited authors, and further enhanced the flexibility and depth of data analysis through RStudio.A tree map was generated using RStudio to enable deeper analysis of keywords. These analyses helped us map the hotspots and trend distributions of the current research landscape and accordingly identify the major development directions within the field.

In addition, we also utilized Microsoft Excel 2019 to conduct statistical analysis and tabulation of high-frequency cited authors, countries/regions, publications, journals, and institutions. Through top-10 lists, we identified authors, institutions, and publications with significant influence in this field, providing valuable references for future research directions and collaboration opportunities.

## 3. Results

**Figure 1.**
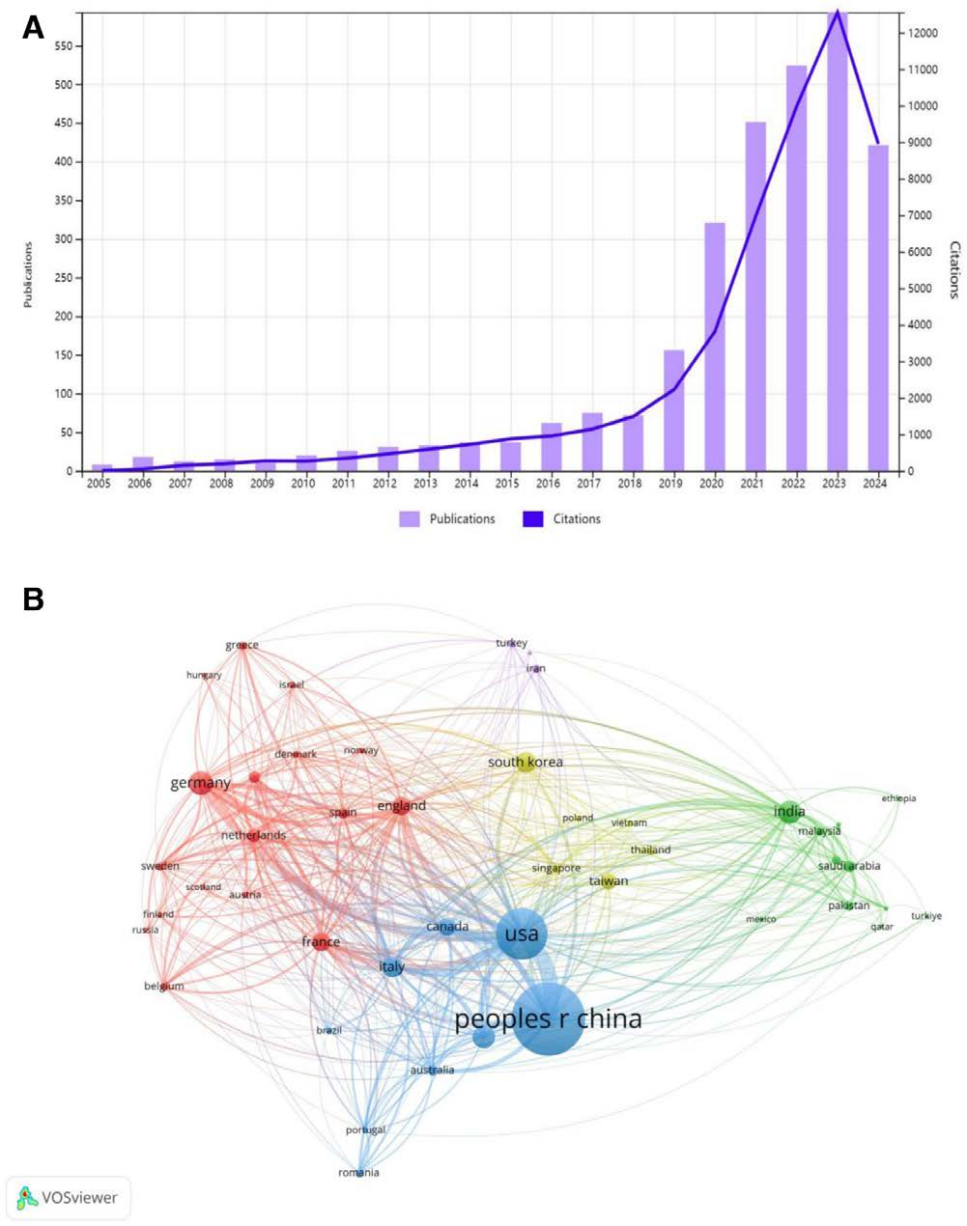
(A) The trend in artificial intelligence in liver cancer publications. (B) Country collaboration network. The thickness of the lines reflects the cooperation strength.

Based on the data retrieval strategy developed in this study, we successfully retrieved and collected a total of 2922 publications from the WoSCC database, spanning from 2005 to 2024. These publications include 2607 research articles and 315 reviews. The data indicates a steady growth in research on AI in the field of liver cancer, with the annual number of publications increasing significantly from 8 in 2005 to 593 in 2023 (Fig. 1A). As of the retrieval date, all publications have been cited a total of 45,601 times, demonstrating a high academic impact. Furthermore, using the h-index as an evaluation metric, the h-index for this research field is 89, and the average number of citations per publication is 17.81. The h-index, as a comprehensive evaluation indicator, effectively reflects the overall performance of researchers, countries, journals, or institutions in terms of both the quantity and quality of academic output, providing an important reference for us to deeply understand the academic development level of this field.

### 3.1. Analysis of productive countries/regions

A total of 103 countries/regions have published academic papers in this field, with the top 10 countries/regions in terms of publication volume are listed in Table 1: China (1226 papers), the United States (681 papers), Germany (191 papers), India (182 papers), Japan (172 papers), Italy (150 papers), South Korea (146 papers), the United Kingdom (125 papers), France (125 papers), and Canada (113 papers). Figure 1B displays the cooperation network among countries/regions, with a minimum publication threshold set at 5 papers. In this figure, the size of the nodes represents the volume of publications, while the thickness of the lines reflects the strength of the cooperation links.

**Table 1:** Top 10 countries/regions with the highest publication volume in AI-related liver cancer research (2005–2024).

Rank	Countries/regions	NP	NC	H-index	Average citation per item
1	PEOPLES R CHINA	1226	17,028	63	14.91
2	USA	681	16,696	64	25.2
3	GERMANY	191	4574	39	24.5
4	INDIA	182	3444	32	19.88
5	JAPAN	172	3464	26	20.69
6	ITALY	150	3355	34	23.16
7	SOUTH KOREA	146	2053	21	14.28
8	ENGLAND	125	2860	32	22.97
9	FRANCE	125	2844	31	23.06

AI = artificial intelligence, NP = number of publication; NC = number of citations.

In terms of total link strength (TLS), The thickness of lines in the country collaboration network (Fig. 1B) corresponds to TLS, which quantifies both: direct collaboration intensity: frequency of coauthored publications between 2 countries. Structural embeddedness: shared partnerships with third-party countries (triadic closure effect) the top 3 countries are the United States (708), China (415), and Italy (282). Extensive cooperation is evident among multiple countries/regions, particularly between China and the United States. As the leading contributors to the literature in this field, China and the United States highlight their prominent positions in research. Additionally, Western European countries such as Italy, the United Kingdom, and Germany demonstrate high levels of participation. Furthermore, Asian countries including India, Japan, and South Korea, as well as other major countries, are present in the cooperation network, indicating significant potential for future development in this field.

Notably, China has the highest total number of citations, reaching 17,028. Meanwhile, the United States ranks first in both the h-index (64) and the average citations per paper (25.2), further underscoring its academic influence in this field. These findings suggest a competitive yet collaborative landscape, with key players driving the progress and innovation in AI research related to liver cancer.

### 3.2. Analysis of productive institutions

**Figure 2.**
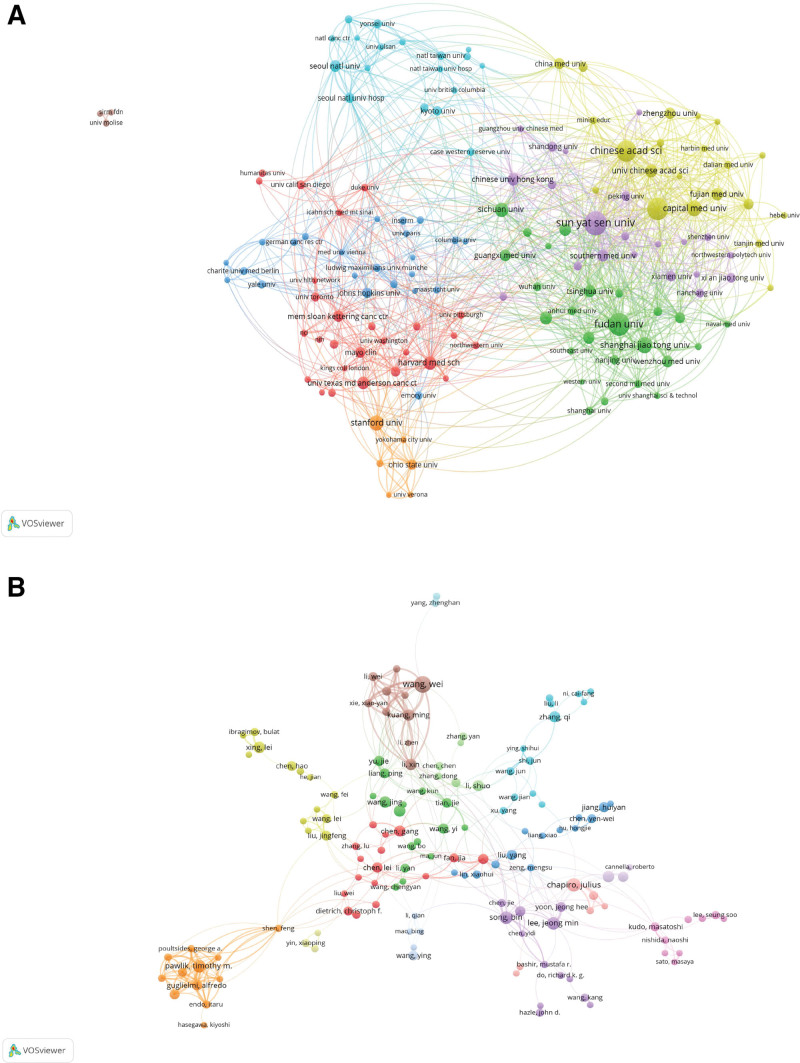
(A) Collaboration network map among institutions, with line thickness reflecting the strength of collaboration. (B) Author collaboration network diagram, with the thickness of the lines indicating the strength of the collaboration.

Over 3991 institutions have contributed to the research field of AI in liver cancer, with 146 institutions publishing 10 or more articles. Table 2 summarizes the top 10 most influential institutions in this field, ranked by the number of publications. The Chinese Academy of Sciences (CHINESE ACADEMY OF SCIENCES) leads the list with 109 publications, followed closely by Sun Yat-sen University (107 publications) and Fudan University (91 publications). In terms of average citations per paper, the University of California System (UNIVERSITY OF CALIFORNIA SYSTEM) stands out with an average of 30.58 citations per article, followed by the Institut National de la Santé et de la Recherche Médicale (INSERM) with an average of 30.39 citations per paper, and Harvard University with an average of 26.58 citations per paper. Figure 2A displays the cooperation network among institutions, revealing the intensity of collaboration in this field. In the ranking of TLS, the top 3 institutions remain the Chinese Academy of Sciences (TLS = 172), Sun Yat-sen University (TLS = 161), and Fudan University (TLS = 125). These data not only reflect the activity and academic contributions of these institutions in the field of AI and liver cancer research but also highlight their central positions in the global research collaboration network. The high level of cooperation among these institutions suggests a collaborative and interdisciplinary approach to addressing the challenges in this research area, which is crucial for advancing knowledge and developing innovative solutions.

**Table 2:** Top 10 institutions with the highest publication volume in AI-related liver cancer research (2005–2024).

RANK	Institutions	NP	NC	Countries/regions	H-index	Average per item
1	CHINESE ACADEMY OF SCIENCES	109	2768	CHINA	29	25.6
2	SUN YAT SEN UNIVERSITY	107	2011	CHINA	23	19.03
3	FUDAN UNIVERSITY	91	1141	CHINA	20	12.66
4	ZHEJIANG UNIVERSITY	90	1182	CHINA	19	13.32
5	UNIVERSITY OF TEXAS SYSTEM	82	1335	USA	18	16.57
6	HARVARD UNIVERSITY	72	1905	USA	23	26.58
7	UNIVERSITY OF CALIFORNIA SYSTEM	72	2194	USA	24	30.58
8	INSTITUT NATIONAL DE LA SANTE ET DE LA RECHERCHE MEDICALE INSERM	61	1836	FRANCE	21	30.39
9	SHANGHAI JIAO TONG UNIVERSITY	53	514	CHINA	13	9.75
10	UNIVERSITE PARIS CITE	52	1365	FRANCE	18	26.5

AI = artificial intelligence, NP = number of publication; NC = number of citations.

### 3.3. Analysis of authors and co-cited authors

This study encompasses a total of 16,958 authors and 61,243 co-cited authors. Table 3 presents the top 10 most prolific authors, with Chapiro, Julius, Izzo, Francesco, and Pawlik, Timothy M. ranking in the top 3 positions, having published 16, 13, and 13 articles, respectively. To visually demonstrate the collaborative relationships among authors, this study utilizes VOSviewer software to create an author collaboration network map (Fig. 2B). In this map, each node represents an author, and the size of the node directly reflects the number of articles published by that author. The lines connecting the nodes symbolize the co-occurrence relationships between authors, while the thickness of the lines further embodies the closeness of these relationships. Generally, highly prolific authors exhibit a higher co-occurrence rate with other authors, indicating their broader collaboration networks within the field.

**Table 3:** Top 10 most prolific authors in AI-related liver cancer research (2005–2024).

Rank	Author	Country	NC	NP	H-index	Average per item
1	Chapiro, Julius	GERMANY	16	719	10	32.26
2	Izzo, Francesco	ITALY	13	227	7	21
3	Pawlik, Timothy M.	USA	13	211	8	16.54
4	Pickhardt, Perry J.	USA	12	484	9	42.5
5	Vilgrain, Valerie	FRANCE	12	98	5	8.33
6	Granata, Vincenza	ITALY	12	222	7	22.33
7	Wang, Wei	CHINA	11	489	10	45.18
8	Tsilimigras, Diamantis I.	USA	11	219	7	20.36
9	Xing, Lei	USA	11	822	10	75.82
10	Petrillo, Antonella	ITALY	11	228	7	24.27

AI = artificial intelligence, NP = number of publication; NC = number of citations.

Co-cited authors refer to the phenomenon where 2 or more authors are cited simultaneously in the same article or multiple articles. This study reveals that a total of 161 authors have been co-cited more than 50 times, demonstrating their significant influence within the field. The top 3 authors in terms of TLS, namely Granata, V. (TLS = 4613), Ronneberger, O. (TLS = 3562), and He, K.M. (TLS = 3319), further confirm their core positions and extensive influence within the field, as evidenced by their high TLS values.

### 3.4. Journals and co-cited journals

Table 4 provides a comprehensive list of the top 10 journals with the highest publication volumes in the field of early diagnosis of hepatocellular carcinoma, along with several key indicators such as normalized publication, impact factor (IF), and number of citations (NC). These indicators offer significant insights for comprehensively assessing the academic influence and contributions of these journals. In terms of publication volume, the top 3 journals are WORLD JOURNAL OF GASTROENTEROLOGY (43), CANCERS (38), and FRONTIERS IN ONCOLOGY (69), which have played pivotal roles in advancing research in this field.

**Table 4:** Top 10 journals with the highest publication.

Rank	Journal	NP	NC	IF (2023)	H-index	Average per item
1	FRONTIERS IN ONCOLOGY	97	823	3.5	14	8.67
2	MEDICAL PHYSICS	73	1213	3.2	20	16.78
3	SCIENTIFIC REPORTS	69	1103	3.8	17	16.1
4	CANCERS	68	661	4.5	16	9.85
5	COMPUTERS IN BIOLOGY AND MEDICINE	50	1053	7	20	21.36
6	EUROPEAN RADIOLOGY	49	1495	4.7	19	31.06
7	DIAGNOSTICS	39	285	3	10	7.38
8	ABDOMINAL RADIOLOGY	38	464	2.3	12	12.39
9	PLOS ONE	38	696	4.7	16	18.37
10	IEEE JOURNAL OF BIOMEDICAL AND HEALTH INFORMATICS	34	534	6,7	12	16.09

IF = impact factor; NP = number of publication, NC = number of citations.

Most of the top 10 journals belong to the medical field. Among them, EUROPEAN RADIOLOGY stands out as one of the most influential journals in this research area, with an NC value of 1495 and an H-index of 19. Meanwhile, IEEE JOURNAL OF BIOMEDICAL AND HEALTH INFORMATICS boasts the highest IF in this field, reaching 12.9.

**Figure 3.**
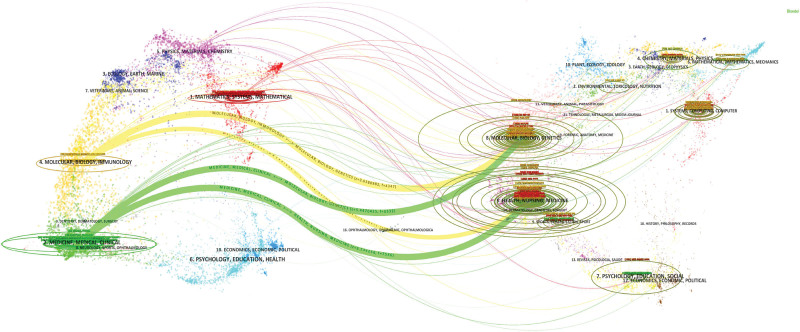
Dual-graph overlay map of journals, with the citation map on the left and the cited map on the right, and curves representing citation path connections.

Co-cited journals refer to 2 or more journals cited simultaneously in the same research work. Figure 3 reveals the complex relationships between citing and cited journals through bimodal mapping. From the perspective of co-citation paths, there are 4 distinct citation paths, and the citing papers mainly concentrate in the following 3 areas: medicine, healthcare, and clinical practice; neurology, sports science, and ophthalmology; molecular biology, biology, and immunology. Correspondingly, the cited papers also primarily focus on the following 3 areas: molecular biology and genetics; public health, nursing, and pharmacy; dermatology, dentistry, and surgery.

### 3.5. Documents citation and co-cited references

**Figure 4.**
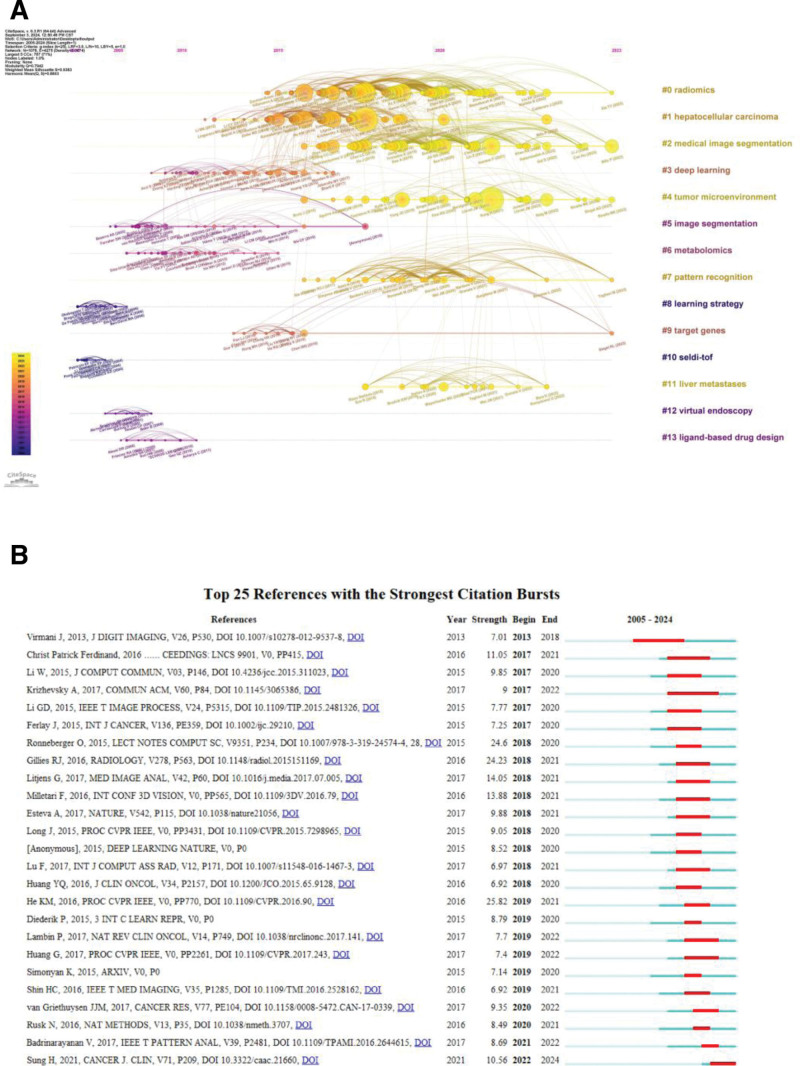
(A) Timeline of co-cited documents. (B) Top 25 references with the strongest citation bursts.

Utilizing VOSviewer software for analysis, the results indicate that a total of 244 articles have been cited more than 50 times. Among these, the top 3 most cited articles are: UNet++: Redesigning Skip Connections to Exploit Multiscale Features in Image Segmentation^[20]^ published on IEEE Xplore, H-DenseUNet: Hybrid Densely Connected UNet for Liver and Tumor Segmentation From CT Volumes^[21]^ also published on IEEE Xplore, and 3D Deeply Supervised Network for Automated Segmentation of Volumetric Medical Images^[22]^ published on ScienceDirect. Among the total of 98,220 co-cited documents, 137 have been co-cited >30 times. The top 3 co-cited articles are Ronneberger et al. (2015, Lecture Notes in Computer Science),^[23]^ Jemal A. et al. (2011, CA: A Cancer Journal for Clinicians),^[24]^ and He and colleagues (2016, Proceedings of the IEEE Conference on Computer Vision and Pattern Recognition).^[25]^ Furthermore, by utilizing CiteSpace software to construct the co-cited document network and conducting cluster analysis (Fig. 4A), this timeline view of co-cited documents, a data visualization method that combines clustering and time slicing techniques. This view not only showcases the distribution of topics within the field through the sequence of cluster labels but also reveals the trends and interrelationships of research topics over time. In the timeline view, nodes of different colors on the same straight line represent documents from different years, with nodes on the left indicating earlier citations and those on the right representing newer citations. Straight lines located at the same horizontal position represent all citations belonging to that cluster, with the cluster label positioned at the rightmost end of the line.

It was found that the co-cited documents can be divided into 14 clusters. The clustering quality indicators, Modularity *Q* = 0.7942 (>0.3) and weight mean silhouette *S* = 0.9383 (>0.5), both indicate that the clustering results are reasonable and highly reliable. On the knowledge map, the first cluster label is “#0 Radiomics,” and the second cluster label is “#1 Hepatocellular Carcinoma.”

In the current timeline view, the clusters closest to the present time include “#0 Radiomics,” “#2 Medical Image Segmentation,” “#4 Tumor Microenvironment,” “#7 Pattern Recognition,” and “#9 Target Genes.” These clusters suggest the current research hotspots and emerging trends in the field.

Finally, an in-depth analysis of the highly cited references was conducted using CiteSpace. Citation Burst Strength in CiteSpace employs the Kleinberg burst detection algorithm to quantify anomalous surges in citation frequency within specific time intervals. Its calculation is based on significant deviations between observed citation counts and expected Poisson distribution values (formula: Burst Strength = [Observed citations ‐ λ(t)]/σ). A high burst strength (typically >5) identifies publications that become core drivers of research fronts within their field.The citation burst phenomenon indicates that certain documents have been widely cited within a specific time period, usually signifying that the research findings in these documents have gained widespread recognition within the field (Fig. 4B). Among the top 25 most frequently co-cited documents, Sung 2021 article titled Global cancer statistics 2020: GLOBOCAN estimates of incidence and mortality worldwide for 36 cancers in 185 countries,^[26]^ exhibits the strongest burst of citations in recent years. This article aims to provide statistical analysis of incidence and mortality data for 36 types of cancer in 185 countries worldwide in 2020, exerting a significant impact on the current research field. The widespread citation of this document underscores its importance in shaping the direction and focus of ongoing research efforts.

### 3.6. Analysis of keywords

Keywords serve as a concise and refined summary of the research content in literature. Their frequent appearance often reflects the research hotspots and trends within a particular scientific field. Additionally, keyword analysis can determine the emergence timepoints of keywords with changing frequencies in the literature network, thereby aiding in defining the boundaries and dynamic changes of the research domain.

**Figure 5.**
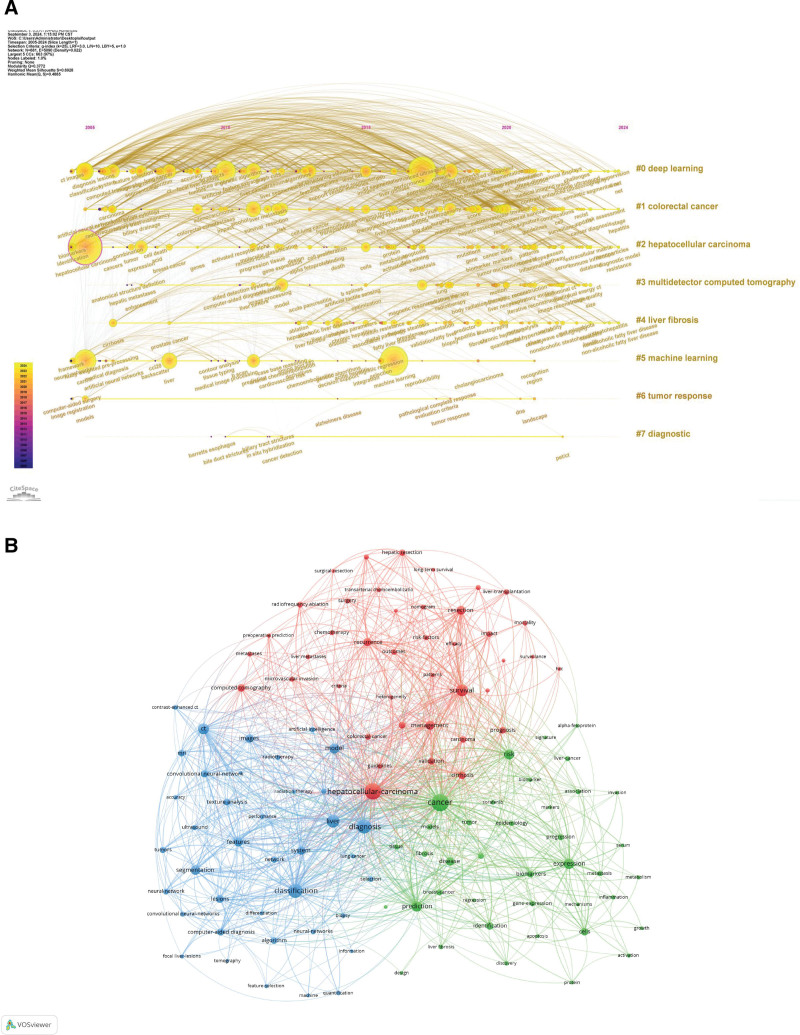
(A) Timeline of keywords. (B) Keyword clustering network map.

In this study, tools such as VOSviewer, CiteSpace, and RStudio were utilized to conduct an in-depth analysis of keywords extracted from 2922 articles. By performing keyword clustering using CiteSpace, we identified the current research hotspots in the field. As illustrated, 8 major clusters have formed, including “#0: Deep Learning,” “#1: Colorectal Cancer,” “#2: Hepatocellular Carcinoma,” “#3: Multidetector Computed Tomography,” “#4: Liver Fibrosis,” “#5: Machine Learning,” “#6: Tumor Response,” and “#7: Diagnostic.” (Fig. 5A). The serial numbers of the clusters do not directly reflect the number of keywords but rather the order of their generation. The size of the nodes is directly proportional to the frequency of keyword occurrences, with larger nodes indicating higher mention counts. In the timeline view, nodes of different colors on the same straight line represent keywords from different years, with left-side nodes indicating earlier keywords and right-side nodes representing newer ones. Each cluster consists of multiple closely related keywords, collectively revealing the internal structure and development trends of the research field.

Between 2005 and 2024, a total of 4419 distinct keywords were used, with 119 keywords appearing more than 20 times. Using VOSviewer, we clustered similar keywords and represented 3 major clusters with red, green, and blue colors (Fig. 5B). The size of the nodes visually reflects the frequency of keyword occurrences. The Table 5 lists the occurrence counts, total link strengths, and topic descriptions of keywords within each cluster, further refining the clustering analysis results. Based on the clustering analysis, we broadly categorize the keywords into 3 clusters: cluster (1) focuses on the application of AI in liver cancer risk assessment; cluster (2) explores the role of AI in liver cancer biology research; and cluster (3) analyzes the value of AI in liver cancer pathology and imaging diagnosis.

**Table 5:** Keywords cluster.

Cluster	Main keywords	Total link strength	Occurrences	Theme description
Cluster 1	Hepatocellular-carcinoma	1048	344	
	Survival	628	186	
	Resection	370	105	
	Management	348	101	
	Recurrence	422	85	
Red	Cirrhosis	249	77	AI in health management of liver cancer
	Computed-tomography	196	74	
	Therapy	191	65	
	Prognosis	197	60	
	Validation	217	60	
Cluster 2	Cancer	1070	388	
	Expression	378	143	
	Prediction	501	142	
	Risk	408	138	
	Disease	237	91	
Green	Identification	224	78	AI in bi ology of liver cancer
	Cells	192	77	
	Biomarkers	168	58	
	Progression	157	49	
	Breast-cancer	110	48	
Cluster 3	Classification	834	270	
	Diagnosis	803	253	
	Liver	539	208	
	CT	458	164	
	Model	367	132	
Blue	Segmentation	356	123	AI in pathology and radiology of liver cancer
	Features	388	101	
	Images	316	98	
	System	327	98	
	Lesions	278	87	

AI = artificial intelligence, CT = computed-tomography.

**Figure 6.**
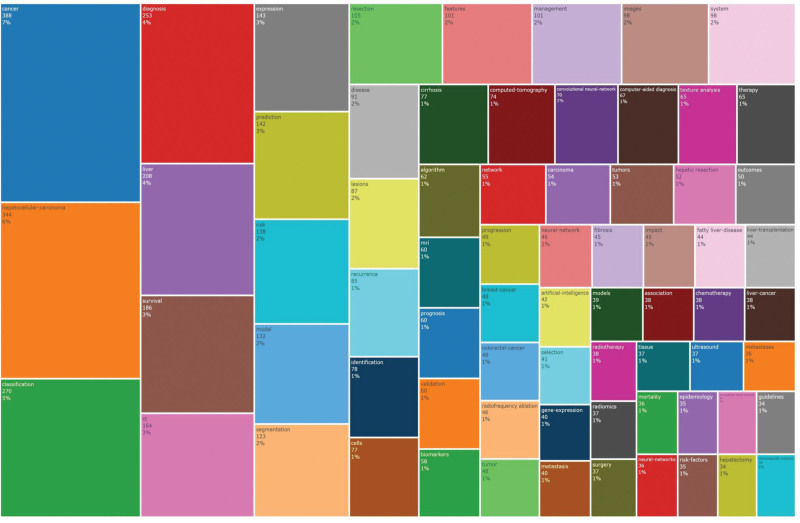
Treemap of keywords.

A deeper analysis of keywords using a treemap (Fig. 6) reveals that, from a data type perspective, the top 3 keywords are “Computed Tomography” (63 times), “Ultrasound” (43 times), and “Magnetic Resonance Imaging” (33 times), reflecting the main trends in data acquisition methods in current clinical research. The objectives of current clinical research primarily concentrate on “Diagnosis” (221 times), “Classification” (181 times), and “Prediction” (118 times), while “Neural Network” (44 times) is the most widely used technical means.

## 4. Discussion

With the continuous advancement of AI technology, its potential in the medical field, particularly in the treatment and research of liver cancer, has become increasingly prominent. This study aims to systematically and quantitatively analyze the current research status of AI technology in the field of liver cancer through bibliometric methods, and to explore the research hotspots and future trends in this area. We selected 2922 relevant articles from the WoSCC database spanning from 2005 to mid-2024 as our data source, primarily utilizing methods such as publication volume statistics, co-occurrence analysis, and keyword analysis.

### 4.1. Discussion of country and institutions

The research results indicate a significant growth in the number of research articles on AI technology in the field of liver cancer over the past decade. Specifically, during the period from 2005 to the second half of 2024, China emerged as the country with the highest publication volume in this field, and also ranked first in total citations. However, the United States led the world in terms of the H-index and average citations per article, demonstrating the broad influence and high quality of its research. In contrast, China’s average citations per article were lower than most countries in the top ten in terms of publication volume, suggesting that Chinese scholars and institutions need to further enhance their research quality and international influence.

Institutional analysis reveals that the CHINESE ACADEMY OF SCIENCES was the most productive institution, highlighting its significant contribution to this field. In terms of collaboration networks, Chinese institutions exhibited good domestic collaboration, while European and American institutions maintained close international cooperation. Chinese institutions should actively seek and strengthen international collaboration to enhance their global perspective and research competitiveness. The top 3 positions in TLS were occupied by the CHINESE ACADEMY OF SCIENCES, SUN YAT-SEN UNIVERSITY, and FUDAN UNIVERSITY, further confirming the leading position of Chinese institutions in this field. Additionally, cases of interdisciplinary collaboration between medical universities and industrial universities demonstrate that the deep integration of medicine and engineering is crucial for promoting the application of AI technology in the medical field, and also emphasize the importance of diversified research team configurations.

### 4.2. Discussion of journals

Regarding journal distribution, although only one of the top ten journals had an IF >5, this reflects the difficulty of publishing research articles on the application of AI in liver cancer in high-level journals. Notably, 8 of the top 10 journals belonged to medical and biological fields, while the other 2 were multidisciplinary journals, indicating a high level of attention from the medical community towards the application of AI in liver cancer research. This finding suggests that when designing interdisciplinary research projects between medicine and engineering, greater emphasis should be placed on integrating and considering the medical perspective to ensure the practicality and clinical value of the research outcomes.

### 4.3. Discussion of co-cited references

“Cluster #0 (Radiomics), #2 (Medical Image Segmentation), and #4 (Tumor Microenvironment) currently represent the most recent co-citation clusters positioned farthest to the right on the timeline visualization, indicating emerging research trends.”

Radiomics refers to a set of techniques for extracting large amounts of quantitative features from medical images, followed by mining these features to retrieve clinically useful diagnostic and prognostic information.^[27]^ Imaging examinations play a pivotal role in the evaluation of various hepatic diseases, including the screening, surveillance, diagnosis, and prognosis of diffuse liver diseases and hepatic tumors. Radiomics has been applied to tumor diagnosis, prediction of microvascular invasion in HCC,^[28]^ surgical resection,^[29]^ HCC prognosis,^[30]^ and radiotherapy for HCC. It has also been applied to predict outcomes in neoadjuvant therapy for colorectal liver metastases.^[31]^ Recent advances in computer science have enabled the clinical application of computer-aided analysis in medical imaging. Among these techniques, radiomics and deep learning represent the most actively researched approaches. Despite involving fundamentally distinct technical processes, both radiomics and deep learning utilize high-dimensional features extracted from images to perform diagnostic and predictive tasks.^[32,33]^

Since 2014, deep learning has demonstrated remarkable performance in image detection and segmentation. Compared to traditional methods, CNNs have proven effective in processing medical images. Notably, fully convolutional networks have achieved exceptional results in medical image recognition, classification, and segmentation.^[34]^

The tumor microenvironment is a complex system comprising diverse cellular and molecular components that collectively shape tumorigenesis, progression, and metastasis. It critically regulates multiple facets of tumor biology, including tumor initiation, advancement, dissemination,and even the establishment of immunosuppressive conditions.^[35]^ Histopathological examination remains the cornerstone for disease diagnosis and prognosis. Advances in imaging technology have established whole-slide imaging scanning of tissue sections as a standard clinical procedure. State-of-the-art whole-slide scanners enable rapid generation of large volumes of whole-slide imagings, capturing histopathological details at high resolution. Consequently, digital pathology is being increasingly adopted in clinical practice and diagnostics.^[36]^ Deep learning algorithms developed for pathological image analysis have been successfully applied to tasks including diagnosis and prognosis prediction.^[37–39]^ These 3 clusters further demonstrate the convergence of recent AI research priorities with HCC investigations.

### 4.4. Discussion of keywords

An in-depth analysis of keyword frequencies revealed that terms such as “classification,” “diagnosis,” and “survival” exhibited high-frequency characteristics in this research field, highlighting the importance of these concepts. In terms of data type applications, CT emerged as the most commonly used data source in AI-assisted liver cancer research, followed closely by magnetic resonance imaging (MRI) and ultrasonography.

According to the current diagnostic guidelines for liver cancer, ultrasonography is recommended as the preferred monitoring tool due to its excellent tolerability and widespread applicability.^[40]^ This technique exhibits a sensitivity ranging from 60% to 80% and a specificity exceeding 90%, demonstrating its effectiveness in diagnosis. Additionally, CT and MRI are also regarded as crucial bases for formulating clinical treatment strategies and staging and classifying tumors in liver cancer patients.^[41]^ Despite the widespread application of ultrasonography in clinical practice, its utilization frequency remains relatively lower compared to CT. This phenomenon is primarily attributed to ultrasonography’s high dependence on the operator’s skills and, in some cases, its insufficient diagnostic accuracy. These factors collectively limit its broader and more in-depth application.

The keywords were grouped into 3 clusters, with research hotspots and trends discussed for each cluster separately.

#### 4.4.1. The role of AI in liver cancer biology research

The biomarker alpha-fetoprotein holds a dominant position in liver cancer detection. Despite its high specificity (typically exceeding 80%), its sensitivity is relatively low (approximately 60%), a characteristic that has been elaborated in related studies.^[42,43]^ Over the past 2 decades, sequencing technology has made significant advancements. By deeply mining vast sequencing data, researchers can construct statistical models to decipher genetic and epigenetic patterns closely related to gene expression and gene products, further predicting the three-dimensional structures of complex and highly specific protein molecules. These technological innovations provide a feasible path for the discovery of early diagnostic biomarkers for liver cancer.^[44,45]^ Moreover, these technological advancements are not limited to liver cancer but also offer robust proof of concept and technical support for extensive screening, early diagnosis, and condition monitoring of human cancers.

#### 4.4.2. The value of AI in liver cancer pathology and imaging diagnosis

In the field of medical image analysis, deep learning has demonstrated consistent and exceptional performance. AI has been extensively applied in CT and MRI imaging to identify liver cancer, exhibiting significant advantages.^[33,46]^ Compared to radiologists, CT-based CNN-assisted diagnostic platforms have not only significantly improved diagnostic accuracy but also effectively shortened diagnosis time, fully demonstrating the good feasibility of AI in clinical applications.^[47,48]^ Compared to radiologists, CT-based CNN-assisted diagnostic platforms have not only significantly improved diagnostic accuracy but also effectively shortened diagnosis time, fully demonstrating the good feasibility of AI in clinical applications.^[21]^ In the field of MRI image segmentation, the application of AI is considered more technically complex than CT image segmentation. Charlie et al showed that CNNs perform comparably to human experts in identifying lesions and even surpass them in some cases. The CNN-based deep learning system achieved an accuracy rate of up to 92% in specific tasks.^[46]^ Additionally, to reduce biases caused by human factors in pathological specimen examinations, AI methods have also demonstrated excellent performance.^[49]^ Huang et al cleverly combined Raman spectroscopy with deep learning to achieve rapid and accurate distinction between tumor tissue and adjacent non-tumor tissue, with a tumor recognition accuracy rate of 82.4%.^[50]^

#### 4.4.3. The application of AI in liver cancer risk assessment

Despite significant progress in liver cancer treatment in recent years, the 5-year survival rate for patients remains only 15% due to diagnostic delays and the limitations of existing treatment efficacy.^[51]^ Therefore, exploring new methods to improve early diagnosis rates, predict patient treatment responses, and survival times for liver cancer is particularly crucial.^[52]^ AI-based liver cancer risk assessment models are increasingly becoming research hotspots. Hao et al designed and validated a predictive model using diagnostic samples obtained from The Cancer Genome Atlas database. This model integrates The Cancer Genome Atlas’s whole-genome methylation data with advanced machine learning methods, deeply assessing the crucial role of DNA methylation in distinguishing tumor tissue from normal tissue in 4 common cancers: breast cancer, colon cancer, liver cancer, and lung cancer. The model demonstrated an accuracy rate exceeding 95%, exhibiting comparable or even superior discriminative ability compared to traditional diagnostic methods.^[53]^ Given the current scarcity of tools for predicting patient clinical prognosis, Wu et al developed and validated a clinical scoring system based on data from the Surveillance, Epidemiology, and End Results Program to assess patients’ overall survival and cancer-specific survival rates.^[54]^ This innovative tool provides valuable insights for clinical decision-making and is expected to have a profound impact on the formulation of future treatment strategies and follow-up studies on liver cancer. To further predict the prognosis of refractory liver cancer, researchers innovatively integrated quantitative image analysis techniques with machine learning and AI technologies.^[55]^ This interdisciplinary approach is expected to provide more precise treatment decision support for clinical practice, thereby improving patient treatment outcomes and quality of life.

### 4.5. Conclusion and future perspectives

Bibliometric analysis reveals a pronounced core-periphery structure within global collaborative networks for AI-driven liver cancer research. Data indicate that African nations exhibit minimal participation in these networks (representing <0.3% of total publications), with the overwhelming majority of affiliated African researchers occupying last authorship positions in internationally coauthored works. This phenomenon suggests that the prevailing Sino-American dominated collaboration paradigm has failed to effectively bridge interregional disparities in knowledge production capacity and may rather exacerbate resource allocation inequities through the Matthew Effect.^[56]^ This polarization phenomenon stems from 3 interconnected structural barriers, *data resource barriers*: peripheral regions face critical shortages of high-quality medical imaging databases (e.g., well-annotated HCC CT datasets), while core nations exhibit minimal sharing of raw data (raw data sharing rate < 15%). *Technological capacity divide*: deep learning model training necessitates high-performance computing capabilities (87% of global TOP100 supercomputing centers are concentrated in China, the US, Europe, and Japan rendering peripheral regions incapable of independently conducting cutting-edge research). *Academic power imbalance*: core-nation institutions dominate research design (including grant applications and methodology selection) in collaborative projects, resulting in scholars from peripheral regions being relegated to “data contributors” rather than “knowledge co-creators.” This structure risks perpetuating “academic colonialism” *extractive knowledge transfer*: core institutions acquire clinical specimens and epidemiological data from peripheral regions through collaborations, yet rarely repatriate research outputs (e.g., patents, proprietary algorithms) to local communities; *impaired sustainable development*: scientific ecosystems in peripheral regions become trapped in a dependency-disempowerment cycle, contravening principles of global health equity.^[57]^

This study uncovers a profound technological preference chasm between AI research and clinical practice in liver cancer diagnostics. Although international guidelines consistently designate ultrasound as the primary screening modality due to its real-time capabilities, low cost, and absence of radiation exposure,^[40]^ our bibliometric analysis reveals a stark inversion in AI research priorities: 45% of imaging-focused AI studies (63/139) publications containing high-frequency keywords) utilize CT data (Fig. 6), while merely 30% engage with ultrasound. This misalignment stems from an algorithmic convenience-first paradigm (CT’s standardized 3D voxel arrays provide native compatibility with mainstream CNNs),^[20]^ whereas ultrasound’s dynamic RF signal processing demands specialized solutions for 3 persistent technical barriers: temporal coherence maintenance across freehand probe movements; in freehand scanning mode, the six-degree-of-freedom pose variations (3 translational axes + 3 rotational axes) of the ultrasound probe induce nonrigid deformation between consecutive frames.^[58]^ Addressing the technical bottleneck of acoustic artifact suppression (which involves eliminating non-biological signal interference arising from ultrasound-tissue interactions to prevent diagnostic misinterpretation) remains critical.^[59–61]^ The current critical bottleneck lies in the cross-vendor data standardization gap. This issue fundamentally stems from divergent hardware architectures among manufacturers, leading to inconsistent imaging manifestations of identical pathological lesions. When AI research diverges from clinical gold standards, its translational outcomes will face fundamental barriers.

Multimodal medical imaging plays a crucial role in the diagnosis and characterization of lesions.^[62]^ However, challenges remain in lesion characterization based on multimodal feature fusion. Despite widespread advocacy for multimodal integration,^[63]^ empirical evidence reveals a stark implementation deficit. Keyword co-occurrence network analysis indicates that <5% of studies achieve genuine fusion of imaging and genomic data. Current approaches predominantly rely on mechanistic concatenation strategies, without constructing cross-modality alignment spaces,^[64,65]^ this direct supervision of multimodality comes from fixed weight coefficients, and the predefined parameters cannot accurately reflect the contribution of different modes. Therefore, current multimodal fusion methods do not consider the relative importance of different modalities and cannot possibly handle the fusion characterization of clinical multisequence images with varying predictive powers.^[63]^

## 5. Limitations

Despite the ability of data visualization analysis using tools such as VOSviewer, CiteSpace, and RStudio to offer a more intuitive understanding of the historical development, current state, and research foci in the field of AI for liver cancer research compared to traditional review methods, this approach also has certain limitations, as outlined below:

1. *Limited data coverage*: Since the analysis relies on specific databases (e.g., WoS CC), papers outside of this database are excluded, making it difficult to comprehensively assess all contributions of AI in liver cancer research.
2. *Language bias*: Given that WoS CC primarily includes English publications, high-quality research in other languages may be overlooked, introducing a language bias.
3. *Limited author contribution identification*: Bibliometric software struggles to accurately differentiate the actual contributions of each author in complex collaborative settings, requiring researchers to assess this through in-depth reading of the original literature.
4. *Lack of author role information*: Software like VOSviewer typically does not directly annotate the identity of the first author or corresponding author when presenting collaboration networks, limiting the depth of analysis to some extent.
5. *Subjective interpretation*: While the analysis process of VOSviewer and similar software is objective, the interpretation of results often depends on the subjective understanding and background knowledge of the researcher, potentially leading to biases in interpretation.
6. *Time lag effect*: Newly published high-quality articles, particularly those with low citation counts (NC) that have not yet accumulated enough citations to be prominently displayed in the visualization, may be overlooked due to the time lag in analysis.

In summary, we anticipate future research to expand data sources to cover more diversified databases and strive to construct a more complete and detailed picture of AI research in liver diseases globally. Additionally, attention should be given to reducing the impact of the aforementioned limitations on analysis results to enhance the comprehensiveness and accuracy of the research.

## 6. Conclusion

This study systematically and deeply analyzed the published literature on AI technology in the field of liver cancer using bibliometric methods. The results indicate that AI technology is developing at an unprecedented rate and demonstrating broad application potential in the diagnosis and treatment of liver cancer. In particular, intelligent analysis of imaging data has become a central and hot topic in current research in this field. However, despite the promising prospects, research on the deep fusion analysis of multisource and multi-type data involved in liver cancer diagnosis and treatment using AI, as well as research on formulating liver cancer treatment decisions based on these data, is still relatively scarce. This indicates an important direction and potential trend for future research.

## Acknowledgments

We would like to thank Department of Medical Engineering Integration and Translational Application Affiliated Hospital of Qinghai University and Dianhua Liu.

## Author contributions

**Conceptualization:** Tingxuan Zhang, Chi Zhang, Weidong Li, Zhanjin Wang.

**Data curation:** Tingxuan Zhang, Youhui Zheng, Zhanjin Wang.

**Formal analysis:** Tingxuan Zhang.

**Funding acquisition:** Tingxuan Zhang.

**Investigation:** Tingxuan Zhang.

**Methodology:** Tingxuan Zhang.

**Project administration:** Tingxuan Zhang, Youhui Zheng.

**Resources:** Tingxuan Zhang.

**Software:** Tingxuan Zhang.

**Supervision:** Tingxuan Zhang.

**Validation:** Tingxuan Zhang, Chi Zhang.

**Visualization:** Tingxuan Zhang.

**Writing – original draft:** Tingxuan Zhang.

**Writing – review & editing:** Tingxuan Zhang, Kaihao Du, Zhan Wang.
